# Using Learner Reviews to Inform Instructional Video Design in MOOCs

**DOI:** 10.3390/bs13040330

**Published:** 2023-04-13

**Authors:** Ruiqi Deng, Yifan Gao

**Affiliations:** 1 Jing Hengyi School of Education, Hangzhou Normal University, Hangzhou 311121, China; r.deng@hznu.edu.cn; 2 Chinese Education Modernization Research Institute (Zhejiang Provincial Key Think Tank), Hangzhou Normal University, Hangzhou 311121, China; 3 College of Civil Engineering and Architecture, Zhejiang University, Hangzhou 310058, China

**Keywords:** MOOCs, videos, video-based learning, learning resources, course reviews

## Abstract

Videos are arguably the most important and frequently used instructional resource in massive open online courses (MOOCs). Recent research has explored learners’ perceptions and preferences regarding MOOC instructional videos. However, these studies are often limited to a small number of specific courses, and few grounded theory studies have been undertaken to investigate this topic. In the present study, a multiple-coder research methodology was adopted to analyze 4534 learner reviews of MOOCs in 14 categories. The study aimed to identify key characteristics associated with learners’ favorable perceptions of MOOC videos, types of supplemental or in-video resources learners perceive helpful to support MOOC video use, and video production features learners value. Results revealed that (a) “organized”, “detailed”, “comprehensible”, “interesting”, and “practical” were the top five important characteristics associated with learners’ favorable perceptions of MOOC videos; (b) learners perceived “presentation slides”, “reading materials”, “post-video assessments”, “embedded questions”, and “case studies” as helpful resources to support their utilization of MOOC videos; and (c) learners found “duration” a more salient production feature than “editing”, “resolution”, “subtitles”, “music”, or “voice”. The findings present implications for MOOC video design and foundations for future research avenues.

## 1. Introduction

Videos are arguably the most important learning resource in massive open online courses (MOOCs). As the primary instructional medium in MOOCs, videos play a critical role in explaining learner satisfaction with the instructional format [1]. Text mining reveals that videos help learners comprehend difficult concepts, address their queries, and make MOOCs interesting [2]. Videos not only help instructors establish a social presence but also contribute to students’ judgement of learning [3]. Learners report high and low learning gains in MOOCs, but they uniformly perceive videos as powerful instructional resources [4]. Additionally, according to clickstream data, videos generate more mouse-click events than any of the other instructional resources on MOOC platforms [5]. The number of videos MOOC learners view is positively correlated with their course performance [6] and completion [7]. Given the importance of videos, this study maintains that the design of instructional videos should be prioritized when developing and revamping MOOCs.

Investigating the key factors associated with users’ favorable perceptions of MOOC instructional videos can help obtain better-informed, richer insights into the design of instructional videos and related learning resources in MOOCs. Recent research has explored MOOC learners’ perceptions and preferences regarding the features of MOOC instructional videos [8]. However, few studies have adopted a grounded theory approach to investigate the key factors associated with users’ favorable perceptions of MOOC instructional videos. These gaps in the research provided the impetus for the collection, processing, and analysis of a corpus of educational big data in order to gain an understanding of learners’ experiences with MOOC videos, thus ensuring better-informed, richer insights into the design of MOOC instructional videos and related learning resources.

## 2. Literature Review

Empirical studies undertaken to provide recommendations on the design of instructional videos in MOOCs can be divided into two broad categories. One research stream analyzes clickstream data to identify users’ interaction patterns while watching MOOC videos [9,10]. Notably, Guo et al. [11] used watching session length as a proxy for engagement, and processed data from millions of video-watching sessions. Based on their findings, they suggested that MOOC instructors should support re-watching and skimming in videos that feature procedural knowledge. Stöhr et al. [6] extracted log files from three MOOCs and observed no differences in video-watching activities between specialists (i.e., individuals with some professional relationship to the course content) and non-specialists (i.e., individuals without formal training in the study area), suggesting that MOOC videos were effective for both specialists and non-specialists. Despite their potential usefulness, clickstream data should be interpreted carefully because they are inferred from user behavior rather than from user responses to specific queries [12,13].

The second research stream employs self-report measures to understand learners’ perceptions and preferences regarding MOOC videos. For example, Mamgain et al. [8] designed a survey to explore learners’ perspectives on Coursera and edX videos. Nishchyk et al. [14] conducted interviews with elderly MOOC users to identify challenges they experienced while watching MOOC videos. However, research in this category often suffers from low sample sizes and/or is limited to a small number of specific courses. To date, few grounded studies on learners’ experiences include a broad selection of MOOC instructional videos. More importantly, the prominent factors related to MOOC learners’ positive experiences with such videos remain elusive and continue to require close examination. This leads to our first research question (RQ1): What key characteristics of MOOC videos are associated with learners’ favorable perceptions of MOOC videos? This question is worth investigating, not only because these contributing factors are insufficiently investigated, but also because the results will provide empirical evidence to guide the improved design of MOOC instructional resources. To add conceptual clarity, this study operationally defines “key characteristics” as terms and expressions MOOC learners used to describe and summarize the distinguishing qualities, attributes, or features of pre-recorded instructional videos.

Although MOOC learning typically revolves around watching instructional videos, it is also dependent on other instructional resources [15]. Through explorations of discrete instructional resources, researchers have generated various recommendations for resource design. For example, Zhang et al. [16] found that quizzes were effective for helping learners identify their strengths, weaknesses, and knowledge gaps and thus recommended that, in order to improve MOOC design, quizzes should be included in MOOCs as a scaffolding strategy. Dang et al. [17] identified lengthy assignments and unrelatable course materials as key barriers to learners’ continued usage of MOOCs and suggested that the removal of these components could contribute to learning success. Although discrete resources have been investigated, little attention has been devoted to how learning resources can be designed to support video-based instruction in MOOCs. This knowledge gap leads to our second research question (RQ2): What resources do learners perceive as helpful to support their use of MOOC videos? It is important to explore the answer to this question because if learners perceive certain instructional resources as helpful, then the design of such resources merits further attention and empirical investigation. To add conceptual clarity, this study operationally defines “resources” as any spoken, written, or visual materials the instructors use to assist MOOC participants in meeting learning expectations.

Scholars have conceptualized video production features as characteristics associated with planning and producing video content, such as showing the instructor’s face on the screen [18]. Research has revealed that certain video production features can positively affect learning processes and outcomes. For example, the presence of pedagogical agents in instructional videos was found to positively affect achievement, regardless of whether agents were designed to gesture [19] or not [20]. Some video production features have been found to have no significant influence on student learning. For instance, no convincing evidence was found that videos shot in authentic settings rather than neutral ones result in enhanced learning performance [21]. Nonetheless, the effects of many video production features on student learning remain inconclusive. For example, while no significant effects of instructor presence were found on learning in Alemdag’s [22] meta-analysis, “talking heads” in video instruction were found to be detrimental to knowledge retention in research by Sondermann and Merkt [23].

Video production features can be expensive or difficult to implement and are moderated by educational contexts. Unlike instructional video users in flipped and traditional classrooms [24], MOOC learners often watch videos in isolation from their classmates and receive limited support from instructors and peers [25]. Video production features proven efficacious in laboratory and face-to-face instructional conditions may not be effective in MOOCs, and vice versa [26]. As such, it is important to know which such production features MOOC learners find helpful prior to deciding whether or not to incorporate them. However, at present, the following research question (RQ3) has not been adequately addressed: What production features do learners value in MOOC videos? This question is important. If MOOC learners repeatedly mention certain video production features, then incorporating and optimizing these features during video production is worthy of consideration. To add conceptual clarity, this study operationally defines “video production features” as the characteristics associated with the process of capturing, editing, and producing instructional videos.

In contrast to capturing the opinions of small numbers of learners who took a specific MOOC, collecting reviews from multiple MOOCs or third-party aggregated MOOC platforms allows for coverage of broad perspectives. Existing MOOC studies have demonstrated the effectiveness of leveraging text-based reviews to obtain insights from large numbers of learners; data collected in this manner avoids the self-selection bias that can be present in data collected through interviews or surveys [27,28,29]. This approach has been used in numerous studies to evaluate learners’ behavior, course acceptance, and study experience. For instance, Deng and Benckendorff [2] processed over 8475 ratings and reviews and determined that the quality of instructional videos was central to users’ positive learning experiences in MOOCs. Wang et al. [30] analyzed student reviews of 18 top-rated MOOCs, revealing that those courses excelled in providing feedback, prompting learners to apply newly acquired knowledge, and drawing instructional resources from real-world settings. Nilashi et al. [31] examined 5668 MOOC reviews and found that course usefulness and the quality of course structure and presentation were vital for learner satisfaction. Similarly, Hew et al. [32] processed reflective statements posted by 5884 MOOC users and discovered that passion and a sense of humor were the most frequently mentioned instructor attributes. To capture learners’ experiences with a wider range of MOOCs and address the research questions, we prepared a large dataset containing learner reviews from MOOCs in 14 categories.

The study makes a valuable contribution to the literature on MOOC instructional videos—the most salient and prevalently used resource in MOOCs—because it collects and considers a large quantity of learner reviews across a broad spectrum of MOOCs, thus constituting a rich source of information for gaining insight into the design of MOOC instructional videos and related resources. The objectives of the current study are to reveal (1) key characteristics associated with learners’ favorable perceptions of MOOC videos, (2) types of instructional resources that learners perceive as helpful and supportive in their use of MOOC videos, and (3) video production features valued by MOOC learners.

## 3. Methods

### 3.1. Data Preparation

Previous research has shown that third-party platforms provide reliable sources of information about learners’ perspectives on the MOOCs they have participated in [2,33]. In the current study, the dataset was collected over two weeks in December 2021 from the largest MOOC platform in China—Chinese University MOOC—which hosts over 10,000 courses from over 700 Chinese higher-education institutions [34]. Python was used to parse webpages and transform Hypertext Markup Language (HTML) into structured data on the course name, course provider, instructor name, and learners’ ratings and text-based reviews. As the information we collected was publicly available online and we performed no interventions during the data-collection process, ethics approval was not required [35]. We collected a total of 1,648,747 MOOC reviews in the following 14 categories: agriculture, arts and history, computer science, design, economics and business, education, engineering, health, languages, law, music and dance, psychology, sciences, and sports.

**Figure 1:**
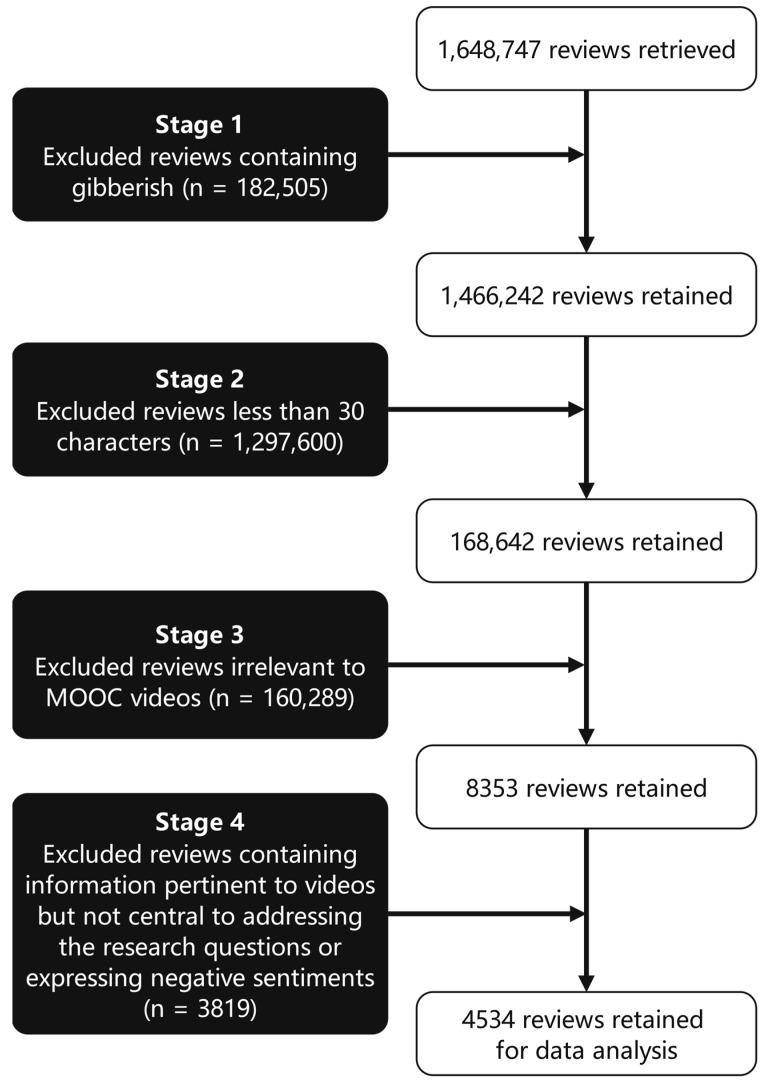
Data pre-processing stages.

We pre-processed the raw data in four stages to improve its quality prior to subjecting it to analysis (Figure 1). To that ensure we analyzed only reviews relevant to MOOC videos and the research questions, after initial evaluation, we excluded 182,505 reviews containing gibberish, unintelligible codes, and the like. Subsequently, we excluded 1,297,600 entries with brief, general comments that were less than 30 characters long and provided little insight into the topic of interest (e.g., the two-word review, “Useful course”). Next, we filtered out the reviews directly relevant to MOOC videos using keywords such as “video” and “lecture”, and excluded the 160,289 entries that remained. Finally, we excluded 3819 reviews that contained information pertinent to MOOC videos but not central to addressing the research questions (e.g., “The last video in Week 2 showed us how to use financial calculators to compute PV…”) or that expressed negative sentiments about MOOC videos. The 4534 reviews comprising the final sample were entered into MAXQDA Analytics Pro. MAXQDA is computer-assisted qualitative data analysis software that can be used for coding and analyzing source materials. This software program has been prevalently used in qualitative educational studies for processing text data [36,37].

### 3.2. Data Analysis

We adopted a grounded method to analyze the data. That is, we did not deliberately impose theoretical frameworks on the data corpus; rather, themes were guided by the research questions and allowed to inductively emerge during the iterative data-analysis process. For example, the following sentence “The videos were interpolated with questions and contributed to knowledge consolidation and reflection.” was coded as embedded questions, because it specifically emphasized interpolating instructional videos with questions.

**Figure 2:**
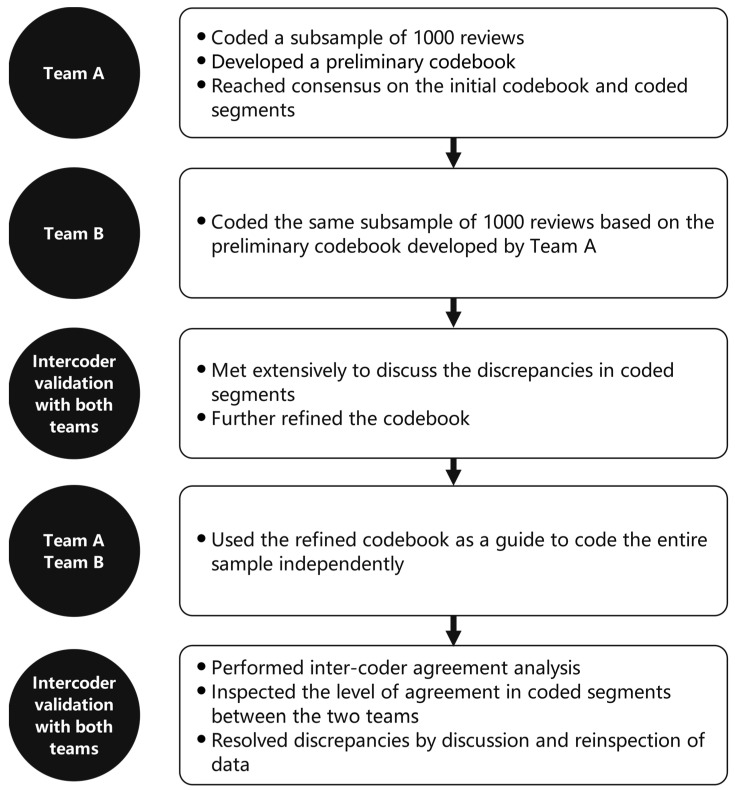
Coding and inter-coder validation processes.

MAXQDA was employed to perform qualitative content analysis. Each sentence was treated as a unit of analysis. The multiple-coder research methodology we adopted in this study was adapted from Corsini et al. [38]. Two teams of research assistants coded the entire sample to ensure the reliability of the results (Figure 2). Team A comprised three research assistants, and Team B comprised two research assistants. All research assistants had backgrounds in educational technology and prior knowledge of and experiences with MOOCs. Firstly, Team A coded a subsample of 1000 reviews and developed a preliminary codebook. The codebook outlined and explained each identified theme. Team A read through the entire subsample together several times, marked the exact beginning and ending of each excerpt, and assigned it a code. The development of the codebook and assignment of the codes were iterative processes. Three members of Team A reached consensus on the initial codebook and the coded segments during repeated offline meetings before handing it over to Team B. Two members of Team B used the codebook developed by Team A to code the same subsample of 1000 reviews, without knowledge of Team A’s coding results. Teams A and B then met extensively to discuss discrepancies between coded segments and further refine the codebook. To strengthen trustworthiness, the refined codebook and coded segments were shared with an expert in MOOC research for constructive feedback and improvement.

Next, Teams A and B each used the refined codebook as a guide to code the entire sample independently. Inter-coder agreement analysis was subsequently conducted in MAXQDA to determine the level of agreement in coded segments between the two teams. The result (87%) indicated a high level of inter-coder agreement. To further improve research reliability, the teams resolved discrepancies by discussion and reinspection of data in face-to-face meetings. Consensus was considered reached when each coded segment was agreed upon by all members of both teams. Analysis results are displayed in the next section by research question. The original reviews were written in Chinese, and we translated partial excerpts into English. The translated excerpts were then checked by a professional English language editing service to ensure accurate usage.

## 4. Results

### 4.1. What Key Characteristics of MOOC Videos Are Associated with Learners’ Favorable Perceptions of MOOC Videos?

In analyzing learner’s reviews, “organized” was identified as an important theme (*n* = 1061), as reflected in the following three aspects. First, learners appreciate video content that explicates the logical relationships between key concepts: “The videos are carefully designed, and the knowledge points and logic are carefully clarified” (Accounting) and “The explanation is very logical…Not only are we told that this phenomenon happens to organisms, but how it was discovered and what it means biologically is also explained” (Molecular Biology). Second, learners appreciate it when the degree of difficulty in videos is elevated incrementally: “The video content transitions reasonably from easy to difficult” (Dance Sport) and “Video content is reasonably arranged, and a step-by-step approach from the easier to the more advanced parts is adopted” (Geophysical Fluid Dynamics). Finally, learners applaud important and difficult information being made prominent in the videos. Two excerpts are illustrative: “The explanations in the video are very clear, and key parts are highlighted in red” (Animation Production) and “The instructor emphasizes key words through use of a heavy tone so that students can easily follow the instructor’s thought process and guidance” (Introduction to Public Finance).

“Detailed” is another important theme identified (*n* = 962). It can be interpreted from two perspectives. On the one hand, learners appreciate it when videos provide detailed, thorough explanations, such as regarding financial knowledge: “The instructor explained investment and financial management information in detail” (Financial Management) and grammar knowledge: “Grammar issues are elaborated on by the instructor” (Chinese Grammar). On the other hand, learners value videos that provide detailed, step-by-step demonstrations, including, for example, software-operation procedures: “When it comes to [teaching] software, demonstrations of the specific operations makes learning easy” (Data Processing in Educational Research); doing yoga: “The decomposition of movements is very precise and detailed” (Fitness Yoga); and dancing “The instructor demonstrated in great detail so that we could master the movements more accurately” (Ballet).

“Comprehensible” is an important theme among learners (*n* = 443). Comprehensible video content is that which is readily comprehensible to the learner, as in the following excerpt: “The explanation in the video is easy to understand. It is not difficult for students with non-related majors to comprehend course content” (Time Value of Money). Learners attribute the comprehensibility of MOOC videos to various factors, including but not limited to the level of detail provided: “The videos elaborate on how to dribble the ball, and they demonstrate the corresponding action for different dribbling methods so that people can easily understand” (Basketball); inclusion of case studies: “Selected cases are used to make the content easier to understand” (Marriage, Career, and Personality); and video segmentation: “Although some of the course content is difficult for me, it becomes easy to understand when broken down into short videos” (Research Methods for Teachers).

Learners identified “interesting” as another theme important to them (*n* = 321). Interesting videos are those that are fun to learn from. Some MOOC instructors use specific communication styles and techniques to generate interest: “Dr Guo is very humorous. Students are not only impressed by his clear logical thinking but also attracted by his ‘funny’ personality” (Fundamentals of Algorithmics) and “The narration is entertaining and humorous, just like a story being told” (Research Methods for Teachers). Other instructors generate interest by designing video content to capture students’ attention, such as by incorporating amusing stories and case studies. The following excerpts illustrate ways in which instructors make learning content interesting: “Each video starts with a lively and interesting short story” (Entrepreneurship Foundation); “Dr Chen discusses many interesting cases in the videos” (Law); “What impressed me most was the personification of the ‘curriculum’ in the form of resumes and interviews, which made dry knowledge vivid and interesting” (Curriculum Design and Evaluation).

“Practical” was an additional important theme identified (*n* = 303). Learners applaud MOOC videos that impart knowledge and skills applicable to their present and future work, life, and study. The following two excerpts illustrate this point: “I learned so much from watching the videos. I learned a lot about how to dress and that body shape and image play an important role in personal impressions and job interviews” (Image Building) and “The examples given in the video reflect what we often come across, and they have helped me solve many problems” (Research Methods for Teachers). Similarly, learners like video explanations and demonstrations that stay close to life: “The content sticks very close to the daily work of the front-line teacher, and the sitcoms in the videos are representative” (Communication Arts for Teachers); “The examples given are relatively contemporary and are similar to everyday life” (Introduction to Public Finance).

### 4.2. What Resources Do Learners Perceive as Helpful to Support Their Use of MOOC Videos?

“Slides” are the most discussed resource supporting videos used in MOOCs (*n* = 456). Learners generally appreciate being provided with slides of the presentations made in the videos: “It is super helpful that the videos are accompanied by the slides” (Botany). Learners found slides helpful when they summarize key information: “The slides are very well organized and summarize the content of the video succinctly” (Entrepreneurship Foundation); provide additional explanations: “The slides accompanying the videos helped me learn better, because they explained the course content in greater detail” (Quality Management); and allow them to revisit the knowledge: “The slides accompanying the videos can help us better review and understand the lesson” (Civil Procedure).

Additionally, “readings” are perceived as helpful resources to support video-based learning (*n* = 363). Typical reading materials include downloadable files and rich texts such as open textbooks, book chapters, and articles. Learners believe that reading materials are useful when they expand horizons: “The best part is that numerous expanded readings accompany the videos, which really broadens your mind” (Pediatrics of Chinese Medicine) and consolidate knowledge: “The MOOC is taught not only in the popular video format but also in text format, which helps me consolidate the knowledge explained by the professor” (Emotion Management).

Learners also perceive “post-video assessments” (*n* = 360) and “embedded questions” (*n* = 38) as helpful. The former refers to computer-graded quizzes and peer assessments learners are required to complete after watching the videos. The latter are multiple-choice and true-or-false questions embedded in the videos. Learners maintain that post-video assessments are helpful because they allow them to assess their learning: “Each chapter contains a quiz, which is conducive to checking our understanding of the lectures” (Accounting) and consolidate knowledge: “The peer-graded assignment gave us opportunities to learn from others’ strong points to compensate for our own deficiencies” (Dance Sport). 

On the other hand, learners believe that embedded questions in MOOC videos are helpful because they encourage active processing of video content: “Interpolated questions can stimulate thinking” (Law); learning assessment: “Inserting quizzes in the lectures worked well, because the questions tested the effectiveness of my learning” (Law); and knowledge consolidation: “The questions embedded in the videos helped me recapitulate what I learned” (Marketing).

Learners point out that “cases”, such as case studies and specific examples, are helpful in-video resources (*n* = 180). Cases are either presented at the beginning of the videos or analyzed throughout. Learners believe that the inclusion of cases is helpful because cases make course content easier to understand: “When the instructor explains a theory that is strange to us, he cites easily understandable cases from life, which is really helpful for understanding the lesson” (Intercultural Communication) and because they generate interest in learning: “Interesting cases are interspersed between relatively boring professional theories to make obscure knowledge come alive” (Law).

### 4.3. What Production Features Do Learners Value in MOOC Videos?

Duration is the most discussed video-production feature (*n* = 313). Learners generally prefer short videos because the brevity allows the focal points to stand out: “The videos are limited to 5 to 10 min, but each moment of content is important” (Pediatric Dentistry); “Sometimes a video covers only a single knowledge point, which makes that point easy to understand” (New Media Theories and Concepts). Moreover, learners note that short videos are helpful in reducing mental fatigue: “The videos are of moderate length and do not cause fatigue” (Journalism) and maintaining attention: “Each video is short, allowing me to stay focused for a long time” (Effective Teaching). Learners also felt that short videos promoted flexible learning: “The duration of each video is not long, so I can make full use of my fragmented time” (Anatomy).

Apart from duration, learners value quality editing (*n* = 106), high resolution (*n* = 86), subtitles (*n* = 51), appropriate music (*n* = 33), and clear voice (*n* = 20) in MOOC videos. Specifically, learners appreciate videos that are professionally edited: “Speaking of video production, the videos are beautifully edited, and the colors and font sizes are just right” (Classical Literature). They also prefer high-resolution videos: “It must be mentioned that the videos in this course are so clear; even the standard definition is extremely clear” (Accounting). This is likely because high-resolution videos improve study experience: “High-definition videos offered better viewing experience” (Logistics) and facilitate practice of the procedural knowledge being taught: “The video is clear, making it easy to imitate the steps” (Learn the Piano from Scratch). 

In addition, learners like captioned videos because the subtitles assist them in notetaking: “The subtitles in the video also play a crucial role in my notetaking” (Classic Poems) and organization of thought: “Some videos have subtitles to clarify the thoughts expressed” (Exercise Physiology).

Finally, learners appreciate videos that feature appropriate background music: “The soundtrack complemented the instructor’s explanation really well” (Wood and Human Civilization) and instructors with clear voices: “The instructor’s voice is clear, so it’s very comfortable to watch the videos” (Software Engineering).

## 5. Discussion

**Figure 3:**
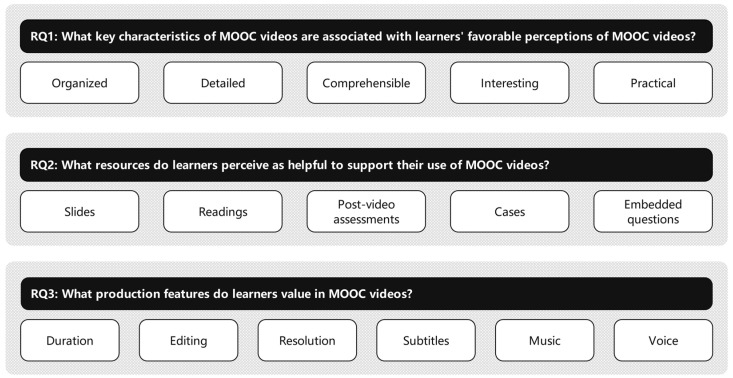
Summary of key findings.

The key findings of this study are summarized in Figure 3. In processing text-based reviews of MOOCs, the present study identified “organized”, “detailed”, and “comprehensible” as important themes associated with MOOC videos. Past research has shown that individuals are likely to recommend a MOOC if it is well-organized [2]. This study adds to the existing body of knowledge on what qualities constitute a well-organized MOOC video in finding that learners appreciate videos that display the logical relationship between key concepts, elevate the difficulty level incrementally, and make important and difficult information prominent. The results partially concur with the observation that displaying the outline of video content was conducive to promoting learners’ grasp of the structural information [39] and learning engagement [40]. Results of this study are also consonant with past research showing that providing simple-to-understand explanations of concepts or procedures is vital in MOOCs [41]. The current study provides preliminary evidence that the understandability of videos can be enhanced through strategies such as providing learners with extensive details and including case studies. Identification of these themes has important implications for practitioners in designing MOOC videos and provides impetus for future research. Investigating antecedent conditions for designing organized, detailed, and/or comprehensible videos for MOOC participants could be of particular interest for other researchers to explore in future research.

This study’s finding that “interesting” is an important theme associated with MOOC videos corresponds with Peng and Jiang’s [42] observation that “interesting” was among the most frequent keywords with which participants evaluated MOOC instruction. Past research has revealed that the learners’ experience of enjoyment, a positive emotion, during MOOCs was positively associated with their lower perceived mental load [43] and higher satisfaction [44] and video engagement [45]. Neutral emotion, however, was found to be negatively correlated with academic performance, suggesting that learners who experienced limited positive emotions while studying obtain poor academic results [46]. According to a recent meta-analysis [47], emotional design features incorporated in multimedia learning can reduce perceived difficulty and enhance learning outcomes, intrinsic motivation, and enjoyment. As MOOCs often revolve around watching videos [1], including emotional design elements in MOOC videos is critical. Moreover, research shows that people can recognize emotions displayed by both human instructors and pedagogical agents in videos [48]. Videos enriched with emotional design elements, such as personalized frame stories, everyday language [49], warm colors, face-like shapes [50], and instructors who display contentedness [51] or happy emotions [52] have been shown to have the potential to facilitate learning. This study provides empirical evidence that learners appreciate MOOC videos that are fun to learn from. Future work could further investigate how the application of emotional design to communication styles and visual elements in MOOC videos can be optimized to facilitate cognitive and affective gains.

This study’s identification of “practical” as an important theme is consistent with recent research showing that MOOC learners emphasize the importance of acquiring knowledge that can be readily transferred to real-life contexts [29]. Mainstream MOOC participants constitute a relatively experienced and qualified segment of the population—individuals who already have bachelor’s or master’s degrees [53]. They often present extrinsic career advancement and professional development motivations for enrolling in MOOCs [54], such as a desire to obtain specific procedural skills in technical fields [55]. Learners typically prefer video demonstrations over paper-based work examples because video demonstrations present information in a more dynamic, step-by-step manner [56]. Research has shown that viewing perspectives play an important role in learners’ acquisition of practical knowledge and skills. For instance, videos alternating between face-to-face and over-the-shoulder views more effectively facilitated mastery of medical procedures than the conventional face-to-face-only or over-the-shoulder-only views [57]. Learners also found first-person perspective videos superior to third-person perspective videos when learning hand-manipulation tasks [58]. Beyond viewing perspectives, future research should continue this line and establish a working set of best practices for designing MOOC videos to facilitate learners’ acquisition of practical knowledge and skills.

This study found that “slides”, “readings”, “post-video assessments”, “embedded questions”, and inclusion of “cases” were among supplemental and in-video resources that users cite as useful in augmenting their experience with MOOC videos. Past research has shown that course resources play a major role in facilitating MOOC engagement [41] and experiences [59]. The evidence obtained in this study adds to existing evidence indicating that lecture slides, readings, post-video assessments, embedded questions, and cases are valuable resources. We contend that the design of these instructional resources should be based on scientific evidence rather than unvalidated assumptions. Providing extra learning materials, such as topics for pre-exam review, has been found to have no significant impact on learning success [60]. Although MOOC instruction is primarily centered around videos, few attempts have been made to investigate how the design of other instructional resources can be engineered to facilitate video-based learning. Future work should move beyond conceptual and theoretical analyses [61] and engage in evidence-based research to generate findings with practical implications for designing slides, readings, assessments, embedded questions, and cases to support video-based MOOC instruction.

This study revealed that embedded questions were perceived as helpful by MOOC learners to diagnose learning, consolidate knowledge, and actively process video content. Previous empirical studies showed that interpolating instructional videos with pop-up quizzes and open-ended questions led to improved academic performance when conducted in the context of credit-bearing online courses [62], flipped classrooms [63], and laboratories [64], but not in the context of MOOCs. It is contended that boundary conditions should be hypothesized as a means of theory development and that multimedia learning researchers should identify boundary conditions for effective video-based learning in MOOCs in order to understand how multimedia design principles work across different educational contexts [26]. MOOC participants often encounter difficulties in comprehending key concepts, leading to disengagement and course abandonment [65]. In attempting to address this problem, it is reasonable to speculate that videos with embedded questions may stimulate MOOC learners to actively engage with instructional materials and that including interspaced breaks may replenish depleted working memory [66]. However, no quantitative research has sought to investigate if the application of embedded questions positively impacts MOOC engagement and outcomes. Such studies would not only clarify potential boundary conditions for designing embedded questions in instructional videos but also reveal the unique benefits of this design strategy to foster student learning in MOOCs.

This study found that compared to “editing”, “resolution”, “subtitle”, “music”, and “voice”, “duration” is the most important production feature to consider when designing instructional videos for MOOCs. MOOC learners often make it less than halfway through videos longer than 9 min [11]. The frequency of video-watching behaviors, such as pausing and seeking, was found to remain stable between 60 and 300 s and to substantially decrease after 300 s [5]. Moreover, recent studies have revealed that total viewing time [67] and learner satisfaction [68] were significantly greater with shorter videos. To date, why learners favor shorter videos has remained largely unknown. Our analysis suggests that learners likely prefer shorter videos due to the prominence of key points and their attentional state being optimized. This observation echoes and helps explain past research showing that students report enhanced focus during shorter videos [69], but start to experience mental fatigue at the 10 min mark when watching videos [70]. Compared to university students, MOOC participants are more likely to view shorter videos in full, and they become considerably more resistant to watching videos the longer they are [71]. Aggregation of these findings highlights the importance of designing shorter videos for MOOCs, as videos are the primary source of instructional content in MOOCs. This is because there are many distractions present around the learners, which can result in them losing focus, particularly those learners who lack self-regulation skills [72,73]. Future research could explore educational interventions to foster video engagement and reduce attention loss beyond designing shorter MOOC videos.

Although potentially important video production features have been identified, we maintain that the effectiveness of these features should be determined in future MOOC research. The effectiveness of video production features may be moderated by environmental factors [26], such as the discipline of study and the learning objectives. For instance, including subtitles in instructional videos was found to positively affect learning performance in the context of second-language acquisition [74,75], whereas it had no effect on immediate comprehension when the topic of the video pertained to stem cells [76]. We suggest that future research investigate the boundary conditions for the video production features MOOC learners value rather than indiscriminately applying these identified features to all MOOC videos.

## 6. Research Implications

The current study provides several implications for practitioners to design instructional videos to support student learning. First, MOOC videos should be “organized”, “detailed”, “comprehensible”, “interesting”, and “practical”. Instructors are advised to present logical relationships between key concepts, incrementally increase difficulty, and make important and difficult information prominent in videos. Videos should provide thorough explanations for declarative knowledge and step-by-step demonstrations for procedural knowledge. It is advisable to use strategies such as providing extensive details and utilizing case studies to improve learners’ comprehension of video content. Additionally, instructors should consider incorporating design strategies that facilitate emotional learning processes and the acquisition of practical knowledge and skills.

Second, MOOC videos should be augmented with supplemental instructional resources, particularly “presentation slides”, “reading materials”, “post-video assessments”, and in-video resources, in particular, “in-video questions” and “case studies”. Instructors could design presentation slides to summarize key information, provide further explanations, and encourage learners to revisit videos to review knowledge. They could provide reading materials that expand horizons and foster knowledge consolidation. Meanwhile, instructors are recommended to design post-video questions and videos with embedded questions to help learners evaluate their learning, consolidate knowledge, and actively process video content. Moreover, instructors are advised to incorporate case studies and examples in videos to facilitate understanding and generate learning interest.

Third, designing shorter videos for MOOCs to reduce learning fatigue and make important information stand out is pivotal. However, simply capturing a video lecture and dividing it into multiple shorter videos is not advisable [77]. Rather, instructors should design each video as a self-contained segment with content that is relatively independent but related to other segments.

## 7. Limitations and Future Directions

This study has several limitations. First, the key characteristics that emerged from the analysis of text-based reviews comprised frequently occurring categories in reviews. While frequency of occurrence is one way to identify salient characteristics, we acknowledge alternative means of conceptualization. Future research could define key characteristics differently or adopt automatic text-mining approaches to investigate this topic [2,78].

Second, we did not control for personal or course characteristics when processing the learner review data. As the course reviewers’ demographic characteristics were unknown, we were unable to analyze the association between learner perceptions of MOOC videos and learner characteristics. Future research is necessary to investigate how MOOC videos and relevant instructional resources can be designed to better cater to learners with certain characteristics or special needs, such as those who use assistive technologies or declare a disability [79]. In addition, the qualitative nature of our research prevents us from performing statistical procedures, such as analysis of variance, to investigate the relationship between course characteristics and user perceptions of MOOC videos. As MOOC participants in different disciplines may exhibit diverse preferences for instructional videos, future research could also control for area of study to gain insights into the design of instructional videos relevant to MOOCs in specific disciplines, such as engineering [80]. Furthermore, it could be beneficial to implement quantitative research to determine discipline-specific effects on learners’ favorable perceptions of MOOC videos. It is expected that such endeavors will provide a more nuanced understanding of instructional video design and users’ instructional-video-design preferences. 

Third, the text-based reviews examined in this study were collected from a Chinese MOOC platform only. These reviews may be influenced by users’ unique national and cultural attributes, and the findings may not be generalizable to the entire population of MOOC participants. Future research should analyze reviews collected from learners in MOOCs outside of China to provide a wider global perspective.

## 8. Conclusions

Instructional videos have become ubiquitous in MOOCs and are integral to teaching and learning in MOOCs. However, they should not be viewed simply as a panacea for enhancing educational performance; suboptimal use of videos can waste students’ time and inflate their judgment of learning [81]. To investigate how to optimize the design of instructional videos and related resources, this exploratory study analyzed a large dataset of reviews and identified key characteristics associated with learners’ favorable perceptions of MOOC videos, types of instructional resources perceived as helpful by learners to support their use of MOOC videos, and video production features valued by MOOC learners. 

The main contributions of this research consist of three key findings and recommendations to be adopted in the design of MOOC instructional videos. First, we discovered that learners favor MOOC videos they perceive as organized, detailed, comprehensible, interesting, and practical. Accordingly, we advise that MOOC videos be designed to characterize the following five attributes: “organized”, “detailed”, “comprehensible”, “interesting”, and “practical”. Second, we also discovered that learners perceive presentation slides, reading materials, post-video assessments, embedded questions, and case studies as helpful to support their use of MOOC videos. Thus, we suggest that MOOC videos should be augmented with supplemental instructional resources, particularly “presentation slides”, “reading materials”, “post-video assessments”, and in-video resources, in particular “in-video questions” and “case studies”. Third, we observed that duration was a more important production feature for instructors to consider than editing, resolution, subtitles, music, or voice when designing MOOC videos. Thus, we recommend that MOOC practitioners design shorter videos for MOOCs to reduce learning fatigue and highlight important information. We hope that the results of the study provide valuable insights into the design of instructional videos and resources to advance MOOC teaching and learning practices.

## Data Availability

The data presented in this study are available on request from the corresponding author.
